# Using long‐term field data to quantify water potential regulation in response to VPD and soil moisture in a conifer tree

**DOI:** 10.1111/nph.70056

**Published:** 2025-03-13

**Authors:** Ibrahim Bourbia, Luke A. Yates, Timothy J. Brodribb

**Affiliations:** ^1^ School of Natural Sciences University of Tasmania Private Bag 55 Hobart TAS 7001 Australia

**Keywords:** drought, iso/anisohydry, modelling, soil moisture, stem water potential regulation, stomatal behaviour, vapour pressure deficit

## Abstract

- The regulation of vascular water potential (Ψ_stem_) by stomata is one of the most dynamic and important behaviours in vascular plants, playing a central role in determining gas exchange and vulnerability to drought. Yet, the species‐specific characterization of Ψ_stem_ regulatory behaviour in response to soil or atmospheric dryness remains elusive.
- We hypothesize that Ψ_stem_ regulatory behaviour can only be defined when the combination of both vapour pressure deficit (VPD) and soil water potential (Ψ_soil_) effects is considered. To test this hypothesis, we collected a high‐resolution time series of Ψ_stem_ using optical dendrometers from trees of a hardy conifer, *Callitris rhomboidea*, monitored across multiple highly variable growing seasons.
- The regulatory behaviour of Ψ_stem_ collected over a total of 571 d could be predicted on the basis of diurnal Ψ_soil_ and VPD (*R* ^2^ = 0.74) using five mechanism‐aligned parameters that describe specific stomatal regulation.
- Our novel approach to predict species‐specific water potential variation in response to seasonal change using data from a continuous Ψ_stem_ monitoring technique creates a new opportunity to quantitatively compare water use and climatic sensitivity between diverse species or genotypes in the field or laboratory.

## Introduction

As plants emerged from swamps and oceans onto the terrestrial earth, a fundamental new pressure came to dominate evolutionary selection in land plants. Water pressure, or more correctly, apoplastic water potential inside the walls of the plant body, is the tension that connects living cells in the plant to water in the soil. This tension enables plants to pull water passively from the soil, but it can also pose a lethal risk when unregulated by the plant. Decreasing soil moisture levels or high rates of transpiration caused by high vapour pressure deficit (VPD) or a combination of both force plant water potential to become more negative, increasing the water tension in the plant body towards damaging values. Under such conditions, the water column in the vascular system begins to break down in a process commonly called xylem cavitation, which occurs as air bubbles are pulled into xylem conduits where they expand, creating embolisms that block the xylem. Xylem embolism disconnects the plant from the soil and has been causally linked to leaf tissue damage and strongly correlated with plant death (Choat *et al*., 2018; Brodribb *et al*., 2021; McDowell *et al*., 2022).

Xylem embolism may be the most obvious damage linked to uncontrolled decline in plant water potential, but other symptoms of excessive xylem tension, such as tissue collapse in leaves (Zhang *et al*., 2016; Corso *et al*., 2020) and roots (North & Nobel, 1991; Cuneo *et al*., 2016; Bourbia *et al*., 2021; Harrison Day *et al*., 2023), also present a clear selection pressure pushing plants to regulate their xylem water potential by the action of stomata. Stomatal pores on the leaf surface enable strong control of transpiration, providing the mechanism for plants to regulate water potential and to restrict soil dehydration. The stringency with which stomata control water potential has been suggested to vary between species and may be an important axis of variation in water use and survival strategy (Gilbert *et al*., 2011; Gholipoor *et al*., 2013; Choudhary *et al*., 2014; Cooper *et al*., 2014; Gleason *et al*., 2022). Because of its potential implications for plant performance and survival during drought, various metrics have emerged that attempt to classify plant species based on the degree of homeostasis in stem water potential (Ψ_stem_) as soil dehydrates (declining soil water potential; Ψ_soil_), such as hydroscape area (i.e. the water potential landscape over which stomata regulate Ψ_stem_) and isohydricity (i.e. the slope of the linear relationship between midday and predawn Ψ_stem_ describing the stringency of stomatal control) (Martínez‐Vilalta *et al*., 2014; Meinzer *et al*., 2016). However, the current view on categorizing species using existing metrics suggests that the mode of Ψ_stem_ regulation is highly unpredictable, with strong variation described within species across seasons (Guo *et al*., 2020, 2024; Kannenberg *et al*., 2022).

One pervasive limitation to the current methods of characterization is that Ψ_stem_ regulation is typically measured as a function of Ψ_soil_ (Martínez‐Vilalta *et al*., 2014; Meinzer *et al*., 2016), while omitting other environmental factors, such as  vapour pressure deficit (VPD), that interact with both Ψ_soil_ and transpiration rate to determine plant hydration (Novick *et al*., 2019, 2024; Grossiord *et al*., 2020; Mencuccini *et al*., 2024). Vapour pressure deficit can strongly modulate the dynamics of Ψ_stem_ independently  of Ψ_soil_, hence influencing the difference between Ψ_stem_ and Ψ_soil_ (Grossiord *et al*., 2020; Bourbia *et al*., 2023; Bourbia & Brodribb, 2024). Increases in VPD typically cause the plant to transpire more water, resulting in lower Ψ_stem_ under constant Ψ_soil_. Therefore, failure to consider the interactive effects of VPD and Ψ_soil_ on Ψ_stem_ regulation will give an incomplete representation of plant responses to water deficits in the soil and atmosphere.

In addition to understanding plant stress exposure, the dynamics of Ψ_stem_ also provide key insight into the regulation of stomatal behaviour. Stomata modulate their opening in response to VPD and Ψ_soil_ by sensing changes in leaf water potential. This, in turn, influences transpiration and the overall regulation of Ψ_stem_ (Schulze & Hall, 1982; Franks *et al*., 1998; Klein, 2014). Therefore, characterizing the specific regulatory behaviour of Ψ_stem_ to the joint effect of VPD and Ψ_soil_ should enable the species‐specific gas exchange to be predicted under all combinations of soil and atmospheric water deficit using hydraulic models. This would provide a more accurate classification of stomatal behaviour than of current metrics relying on soil moisture alone (Martínez‐Vilalta *et al*., 2014; Meinzer *et al*., 2016).

In this study, we aim to examine how VPD and Ψ_soil_ interact with plant behaviour to regulate Ψ_stem_. We hypothesize that Ψ_stem_ regulatory behaviour of a tree species could be precisely captured over multiple highly variable growing seasons when the combination of both VPD and Ψ_soil_ is considered. To test this hypothesis, we collected a fine‐scale time series (15–30‐min intervals) of *in situ* Ψ_stem_ monitored with optical dendrometry in field‐grown specimens of the conifer species *Callitris rhomboidea*, enabling us to derive a mathematical model that captures Ψ_stem_ regulation over 4 yr of highly variable climatic conditions. We then use the parameters of the model to quantify stomatal regulation in response to the joint effect of VPD and Ψ_soil_.

## Materials and Methods

The experiment was carried out in Pelverata located on the southeast of Tasmania, Australia (43°03′03.5″S 147°06′15.8″E, 250 m elevation). The local climate is characterized by cool, wet winters and relatively mild summers with episodic rainfall events and maximum daily air temperatures ranging between 22°C and 30°C. The soil on the site consists of well‐drained, clay loam soils.

### Continuous monitoring of Ψ_stem_

Ψ_stem_ was continuously monitored on four 12‐yr‐old *C. rhomboidea* R. Br. Ex A. Rich (Cupressaceae) trees (5 m tall) over multiple highly variable growing seasons across a 4‐yr period between 2021 and 2024 using optical dendrometers (Bourbia *et al*., 2021, 2023; Bourbia & Brodribb, 2023; Supporting Information Table S1).

The trees were separated by an average distance of 50 m. In each tree, an optical dendrometer was attached to a determinate nongrowing branchlet (< 3 mm in diameter) to continuously monitor its width changes at 15–30 min intervals. Branchlet width was calibrated in each individual tree against Ψ_stem_ measured periodically (every 15–20 d) during the study period with a Scholander pressure chamber (PMS Instruments, Corvallis, OR, USA). These measurements were performed on neighbouring nontranspiring leaves that were wrapped in a damp paper towel and aluminium foil for at least 1 h before measurements. The covered leaves were then excised with a razor blade and placed in a sealable bag with moist paper towels. They were then put in a dark box, transported to the laboratory and measured within 1 h after the leaves were collected. Plant‐specific relationships between branchlet width and measured Ψ_stem_ using the pressure bomb were established and used to determine Ψ_stem_ dynamics during the experimental period (Fig. S1).

Ψ_stem_ measurements were paired with site‐level air temperature and humidity that were monitored continuously at 15–30 min intervals throughout each season using an SHT31 weatherproof temperature and humidity sensor installed within the mid‐canopy of one of the trees to capture the microclimate surrounding the transpiring leaves. Leaves of *C. rhomboidea* are needles, resulting in a small boundary layer around them. This means that the VPD measured within the canopy should closely correspond to the actual VPD at the leaf level. These measurements were used to calculate VPD (Buck, 1981). Rainfall data were obtained from the Groove weather station maintained by the Australian Bureau of Meteorology (Station ID: 94220) located *c*. 6 km from the field.

**Fig. 1:**
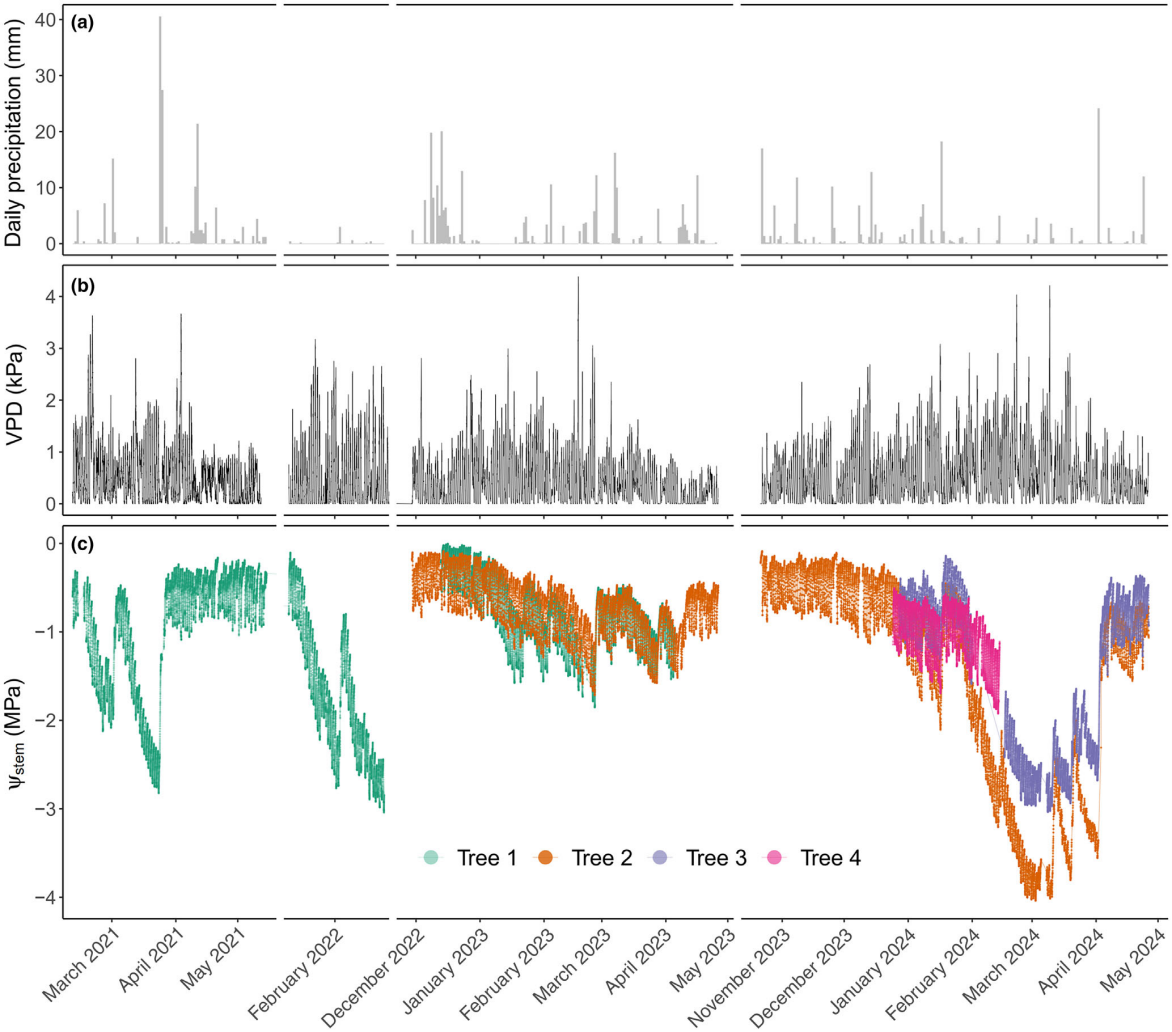
Time series of (a) daily precipitation, (b) diurnal variation in vapour pressure deficit (VPD) and (c) *in situ* measurements of diurnal stem water potential (Ψ_stem_) monitored at 15‐ to 30‐min intervals with optical dendrometers in four individual trees of *Callitris rhomboidea* across multiple growing seasons over a 4‐yr period between 2021 and 2024.

The four trees were not monitored for the same duration or concurrently for most of the study period (Fig. 1). Two trees were monitored over two and three growing seasons, while the other two were monitored for only one growing season. However, we view the asynchronous sampling as an opportunity to test our water potential behavioural model's validity and generalizability (as mentioned in the modelling part described later).

### Characterizing the regulatory behaviour of ΔΨ to VPD and Ψ_soil_

To capture the long‐term variation in the soil‐to‐stem water potential difference (ΔΨ) regulation in response to changes in VPD and Ψ_soil_, we constructed a model of ΔΨ using a single relatively steady‐state maximum daytime ΔΨ to represent each day. We chose steady‐state daily maxima instead of diurnal ΔΨ to avoid the potential confounding effect of capacitance on diurnal ΔΨ dynamics that can occur under non‐steady‐state conditions of Ψ_stem_. Steady‐state conditions refer here to conditions where Ψ_stem_ reached a minimum value during daytime and remained relatively constant (< 5% change) for at least 1 h. In each individual tree, maximum daily ΔΨ was calculated as the difference between minimum mean daytime Ψ_stem_ (most negative value) averaged over a 2‐h period (using 4–8 datapoints) and its corresponding daytime Ψ_soil_ (Fig. S2). The corresponding daytime Ψ_soil_ throughout the study period for each individual tree was determined using linear interpolation between the optically derived mean predawn Ψ_stem_ values (maximum value of Ψ_stem_ before sunrise averaged over 2 h). Under conditions of significant nocturnal transpiration due to high nocturnal VPD, predawn Ψ_stem_ does not represent Ψ_soil_ due to incomplete equilibration (Sellin, 1999; Kavanagh *et al*., 2007; Kangur *et al*., 2020). For this reason, predawn Ψ_stem_ values used for interpolating daytime Ψ_soil_ in each individual tree throughout the study period were selected on the nights when humidity remained above 95% (VPD < 0.05 kPa) throughout the whole night (between midnight and before sunrise; Fig. S2). Under such conditions, night‐time transpiration was found to be negligible in this species (Bourbia & Brodribb, 2024); hence, predawn Ψ_stem_ was assumed to have been given sufficient time to establish equilibrium with Ψ_soil_ in the rooting zone. However, it is important to note that while the interpolated daytime Ψ_soil_ corresponding to the minimum daytime Ψ_stem_ used to calculate ΔΨ can accurately reflect the Ψ_soil_ at the root surface in relatively well‐hydrated soils, it may not fully capture this during soil drying, particularly during daytime transpiration. During soil drying, the water potential at the soil–root surface interface can decline alongside Ψ_stem_ during daytime transpiration due to a potential drop in rhizosphere hydraulic conductance (Carminati *et al*., 2013; Wankmüller *et al*., 2024). Hence, using predawn Ψ_stem_ or interpolated Ψ_soil_ can lead to an overestimation of daytime Ψ_soil_ corresponding to minimum daytime Ψ_stem_ and, consequently, the magnitude of ΔΨ during soil drying. Therefore, caution is required in future studies to account for this potential inaccuracy. This could be resolved if the dynamics of rhizosphere hydraulics (soil–root surface hydraulic conductance) were measured, which was not addressed in the current study. However, it is important to emphasize that this does not alter the overall pattern of ΔΨ regulation but rather affects only the extent of the response; hence, it can still be valid when comparing species grown in the same soil type. Furthermore, under relatively hydrated conditions, soil‐to‐root hydraulic conductance remains constant under changing daily transpiration even at high VPD.

We analysed the relationship between maximum daytime ΔΨ collected for a total of 571 d and environmental variables VPD and Ψ_soil_ (Fig. 3, to be described later). Our statistical model used prior knowledge based on the principles of Darcy's law explaining water flow and generic stomatal regulation behaviour in plants (Grossiord *et al*., 2020) to constrain the fitting process to the following exponential relationship between ΔΨ and VPD:

(Eqn 1) $$\Delta \Psi =\theta \left(1-e^{-\lambda \times \mathrm{VPD}}\right)+\varepsilon$$

where θ and λ characterize the asymptote and rate of curvature, respectively, and $\varepsilon \sim N\left(0,\sigma^{2}\right)$ is the distribution of residual errors. The shape of this two‐parameter curve ranges from an approximately linear trend (low λ, implying low sensitivity of stomata to VPD) to a highly nonlinear relationship characterized by a rapid initial increase followed by stabilization (high λ, implying high sensitivity of stomata to VPD). A representative set of curves for the studied species is shown by the lines in Fig. 4(a) (to be described later). This model is able to accommodate stomatal responses observed in most species, where stomata conductance declines exponentially with increasing VPD (Grossiord *et al*., 2020). However, in some species, stomatal conductance may remain insensitive to low VPD up to a certain threshold, after which it declines exponentially (Franks, 2004). In such cases, ΔΨ would initially increase proportionally (i.e. linearly) with VPD, and the slope of its increase would decline (i.e. transition into a nonlinear model) after the threshold is reached (Bourbia *et al*., 2023; Sharma *et al*., 2024). An appropriate generalization of this model, to account for species with an initially insensitive VPD response, would be to model a linear response (intercepting zero) for the insensitive region, with a changepoint into an exponential‐shaped model of the form presented in Eqn 1 for the remaining VPD values.

Next, we extend the base model (1) to allow both θ and λ to depend on Ψ_soil_, which we write as.

(Eqn 2) $$\begin{array}{c} \theta =\left\{\begin{array}{c} \theta_{0}\;\Psi_{\text{soil}}\geq \Psi_{\text{soil}\_\text{threshold}}\\ \theta_{0}e^{-\alpha (\Psi_{\text{soil}\_\text{threshold}}-\Psi_{\text{soil})}}\;\;\Psi_{\text{soil}}<\Psi_{\text{soil}\_\text{threshold}} \end{array}\right. \end{array}$$

and

(Eqn 3) $$\begin{array}{c} \lambda =\left\{\begin{array}{c} \lambda_{0}\;\;\Psi_{\text{soil}}\geq \Psi_{\text{soil}\_\text{threshold}}\\ \lambda_{0}e^{-\beta (\Psi_{\text{soil}\_\text{threshold}}-\Psi_{\text{soil})}}\;\;\Psi_{\text{soil}}<\Psi_{\text{soil}\_\text{threshold}} \end{array}\;\right. \end{array}$$

These models set θ and λ to the estimated (constant) values $\theta_{0}$ and $\lambda_{0}$, respectively, for values of Ψ_soil_ above (less negative than) the estimated soil water potential threshold (Ψ_soil___threshold_) and specify an exponential change thereafter for increasing negative values of Ψ_soil_. Under increasing drought conditions, every plant will eventually close its stomata completely; therefore, the model for θ must tend towards zero at very negative Ψ_soil_. However, we do not necessarily expect a continuous decline in θ as Ψ_soil_ begins to decline from the saturated conditions. Rather, it is likely that there exists a range of Ψ_soil_ conditions where θ and λ are effectively constant, characterising a stable relationship between ΔΨ and VPD (Franks *et al*., 2007). It is important to note that while the model allows for a threshold in Ψ_soil_, it also includes the possibility of no threshold as a limiting case and thus tests the hypothesis of a stable relationship between ΔΨ and VPD under changing Ψ_soil_. The reason for using an exponential decay in Eqns 2, 3, rather than a linear trend, is to ensure that both θ and λ remain positive. For small values of $\Psi_{\text{soil}\_\text{threshold}}-\Psi_{\text{soil}}$, α and β, the trends are, in any case, approximately linear. Examples of these trends for θ and λ are shown in Fig. 4(b,c) (to be described later), respectively, where the rate α is positive valued (i.e. θ decreases below the threshold) and the rate β is negative valued (i.e. λ increases below the threshold).

Finally, to allow us to include observations for different plants across different growing years, we used a hierarchical structure to estimate $\theta_{0}$ and $\lambda_{0}$ as correlated normally distributed random effects with separate levels for each plant–year combination.

The model was fit with the R package brms (Bürkner, 2017) for full Bayesian inference using Hamiltonian Monte Carlo sampling methods. The estimated posterior comprised four chains of 2000 iterations, and model convergence was established using the improved $\hat{R}$ statistic (Vehtari *et al*., 2021) and visual inspection of the chains. We also computed leave‐one‐out (LOO) cross‐validation predictive distribution to provide an estimate of the LOO *R* ^2^ value for the model (Vehtari *et al*., 2017). The associated LOO distribution and *R* ^2^ value rigorously assess the model's ability to generalize by estimating the predictive likelihood on withheld data. Further diagnostics included plots of the residuals against both VPD and Ψ_soil_ and posterior‐predictive plots, which are provided in Figs S4, S5.

### Modelling *g*_c_ behaviour from ΔΨ

Based on the application of physical laws of diffusion and fluid flow (Whitehead, 1998), it is possible to reconstruct the whole plant stomatal response to environmental factors (Ψ_soil_ and VPD) from observed ΔΨ using the following equation:

(Eqn 4) $$g_{c}\propto \frac{K_{s-p}\times \;\Delta \Psi \;}{\mathrm{VPD}}$$

where *g*_c_ is whole canopy diffusive conductance, and *K*_s‐p_ is soil‐to‐leaf hydraulic conductance. In this case, *K*_s‐p_ was set constant across the entire range of Ψ_soil_ experienced by the trees. Although this simplifying assumption is likely to be violated under conditions of severe water deficit (North & Nobel, 1991; Rodriguez‐Dominguez & Brodribb, 2020; Bourbia *et al*., 2021), we considered that introducing the complexity of a Ψ dependency of *K*_s‐p_ was unlikely to significantly change the predicted stomatal behaviour in *C. rhomboidea*. This is because *K*_s‐p_ in this species has been consistently reported to remain stable under diurnal changes in transpiration and during the early stages of soil drying, declining only after Ψ_soil_ drops below −1 MPa (Bourbia *et al*., 2021, 2022; Bourbia & Brodribb, 2024). This threshold coincides with the Ψ_soil_ threshold beyond which the asymptote (θ) (Eqn 1) was found to decrease sharply due to strong stomatal closure in the studied species (see the Results and Discussion section). Therefore, based on Eqn 4, the impact of declining *K*_s‐p_ on predicted *g*_c_ in the Ψ_soil_ range below −1 MPa is rather minimal and would only increase the already steep slope of the closure response caused by Ψ_soil_ in this region. *g*_c_ was normalized to the maximum value and expressed as a percentage of the maximum.

## Results and Discussion

To achieve our aim of characterizing Ψ_stem_ regulation in whole plants in response to VPD and Ψ_soil_ over multiple seasons and years, it was necessary to collect a substantial data series that faithfully recorded Ψ_stem_ at high temporal resolution. The data collected from four trees over 4 yr using optical dendrometers provided the necessary data set, with over 64,962 individual records of Ψ_stem_ collected between 2021 and 2024 (Fig. 1). Throughout the study period, the monitored trees exhibited strong variation in diurnal Ψ_stem_ and experienced a wide range of VPD and Ψ_soil_ (predawn Ψ_stem_) that was associated with seasonal variation in VPD, episodic drought and rainfall events (Fig. 1). The four trees experienced relatively similar variations in maximum daytime VPD and temperature during the measurement period across the four seasons. Mean daytime air temperature and VPD ranged from 10°C to 32°C, and 0.34 to 4.09 kPa, respectively. Different trees appeared to have had access to different soil moisture levels during soil drying, as shown by differences in predawn Ψ_stem_ experienced throughout each growing season, likely due to variability in root depths between trees (Fig. 1). For instance, during the growing season of 2024, the monitored trees were all hydrated to the same level (Ψ_soil_ close to 0 MPa) in the beginning of the season, but as the drought progressed, Ψ_soil_ experienced by the trees diverged considerably, ranging from −2.6 to −3.6 MPa during the driest period. A degree of variation in root depth or local soil water supply was expected, considering that trees were separated by distances of 50 m and soil depth may have been variable. The trees also experienced different soil moistures between different years due to variation in precipitation. For example, the Year 2023 was exceptionally wet compared with other years, as predawn Ψ_stem_ remained high and above −1 MPa throughout the entire season, whereas the Year 2024 was the driest, with predawn Ψ_stem_ reaching a minimum between −2.6 and −3.6 MPa. This heterogeneity in soil water availability within and between years provided an additional source of variation that provided an ideal test of our general behavioural model for the species.

### Diurnal regulation of ΔΨ

**Fig. 2:**
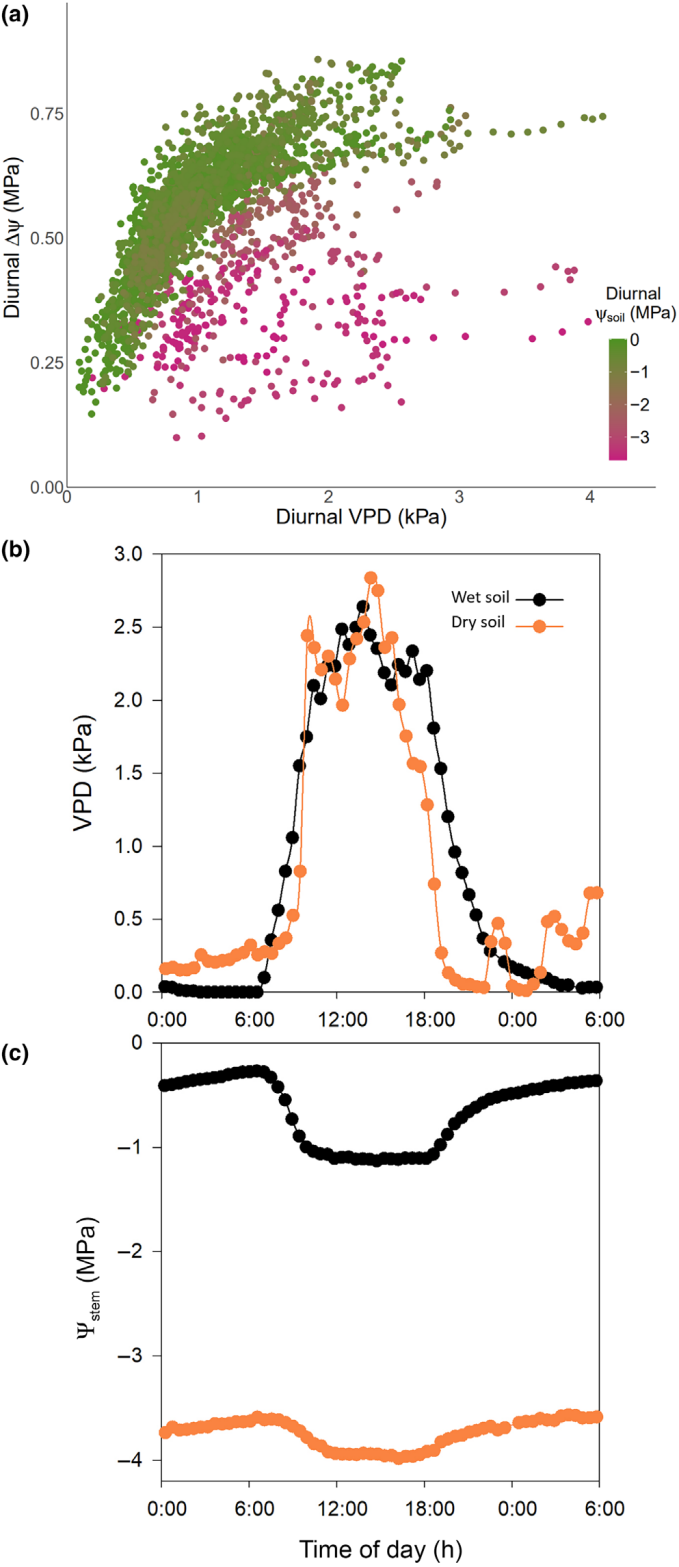
Response of diurnal soil‐to‐stem water potential difference (ΔΨ) to vapour pressure deficit (VPD) and soil water potential (Ψ_soil_). (a) Response of diurnal changes in ΔΨ to corresponding diurnal changes in VPD and Ψ_soil_ between 11:00 h and 16:00 h in one tree (Tree 2) of *Callitris rhomboidea* monitored over two highly variable growing seasons of 2023 and 2024 (see the remaining three individual trees in Supporting Information Fig. S3). (b) Example of diurnal pattern of stem water potential (Ψ_stem_) in response (c) to changes in diurnal VPD over 1 d in one representative tree of *C. rhomboidea* under wet (black closed circle) and dry soil conditions (orange closed circle).

We restricted the analysis of diurnal ΔΨ to the daytime periods between 11:00 and 15:00 h, where stomatal opening was not impacted by light limitation or the circadian effects due to the time of day; hence, the dynamics of diurnal ΔΨ were expected to be influenced mostly by the response of stomata to VPD and Ψ_soil_. The within‐day patterns of Ψ_stem_ variation exhibited a saturating relationship between ΔΨ and VPD, where ΔΨ increased with increasing VPD, then approached saturation as VPD exceeded 1 kPa (Figs 2a, S3). Such a relationship would be expected if stomatal conductance responded with the typical 1/VPD dependence expected under most stomatal control models (Oren *et al*., 1999; Grossiord *et al*., 2020). This relationship, however, appears to be influenced by Ψ_soil_ as well (Fig. 2a), as evidenced by a substantial reduction in the magnitude of ΔΨ response to VPD under dry soils (more negative Ψ_soil_) compared with well‐hydrated soils (Ψ_soil_ close to 0 MPa; Fig. 2b,c).

**Fig. 3:**
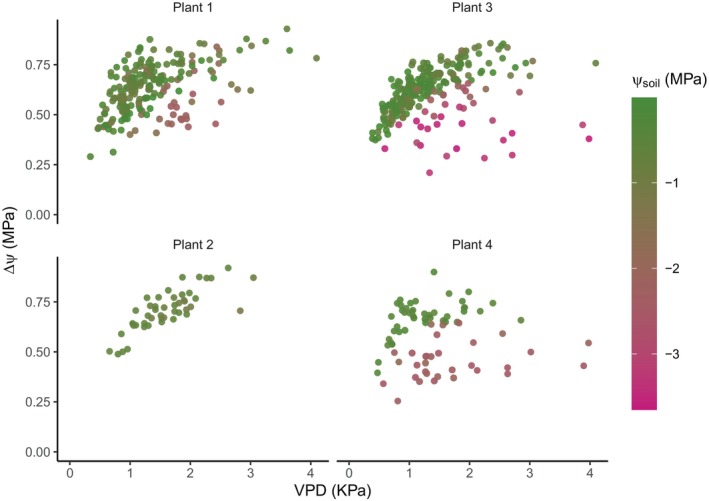
Response of daytime maximum soil‐to‐stem water potential difference (ΔΨ = Ψ_soil_−Ψ_stem_) to its corresponding maximum daytime vapour pressure deficit (VPD) under variable soil water potentials (Ψ_soil_) monitored continuously and *in situ* in four individual trees of *Callitris rhomboidea* across multiple highly variable growing seasons over 4 yr between 2021 and 2024.

To investigate these patterns further, the time frame of observation was changed from minutes to days, using only one steady‐state daily maximum ΔΨ per day. We chose steady‐state daily maxima instead of diurnal ΔΨ to avoid the potential confounding effect of capacitance on diurnal ΔΨ dynamics under non‐steady‐state conditions of diurnal VPD. When ΔΨ response to Ψ_soil_ and VPD was expressed in terms of a single daily maximum, all trees showed a very similar trend to that observed using the diurnal time series (Fig. 3). Hereafter, ΔΨ and VPD will refer to their steady‐state maximum daily values unless stated otherwise.

### Relationship between ΔΨ and VPD under wet conditions

**Fig. 4:**
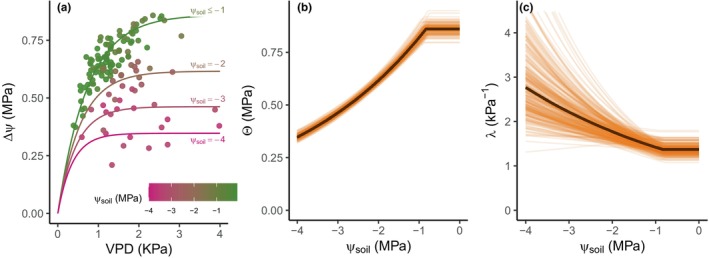
Response of maximum daytime soil‐to‐stem water potential difference (ΔΨ) to vapour pressure deficit (VPD) and soil water potential (Ψ_soil_). (a) Response of daytime maximum ΔΨ to its corresponding maximum daytime VPD as Ψ_soil_ becomes more negative due to soil drying in one representative tree of *Callitris rhomboidea* from one growing season (Year 2024). The curves show the expected value of the modelled response as a function of VPD and Ψ_soil_. (b) and (c) show the change in the mean (black line), and 200 posterior samples (yellow lines) of the asymptote θ and the rate λ of the asymptotic relationship between ΔΨ and VPD as Ψ_soil_ become more negative due to soil drought (see Eqn 1).

**Fig. 5:**
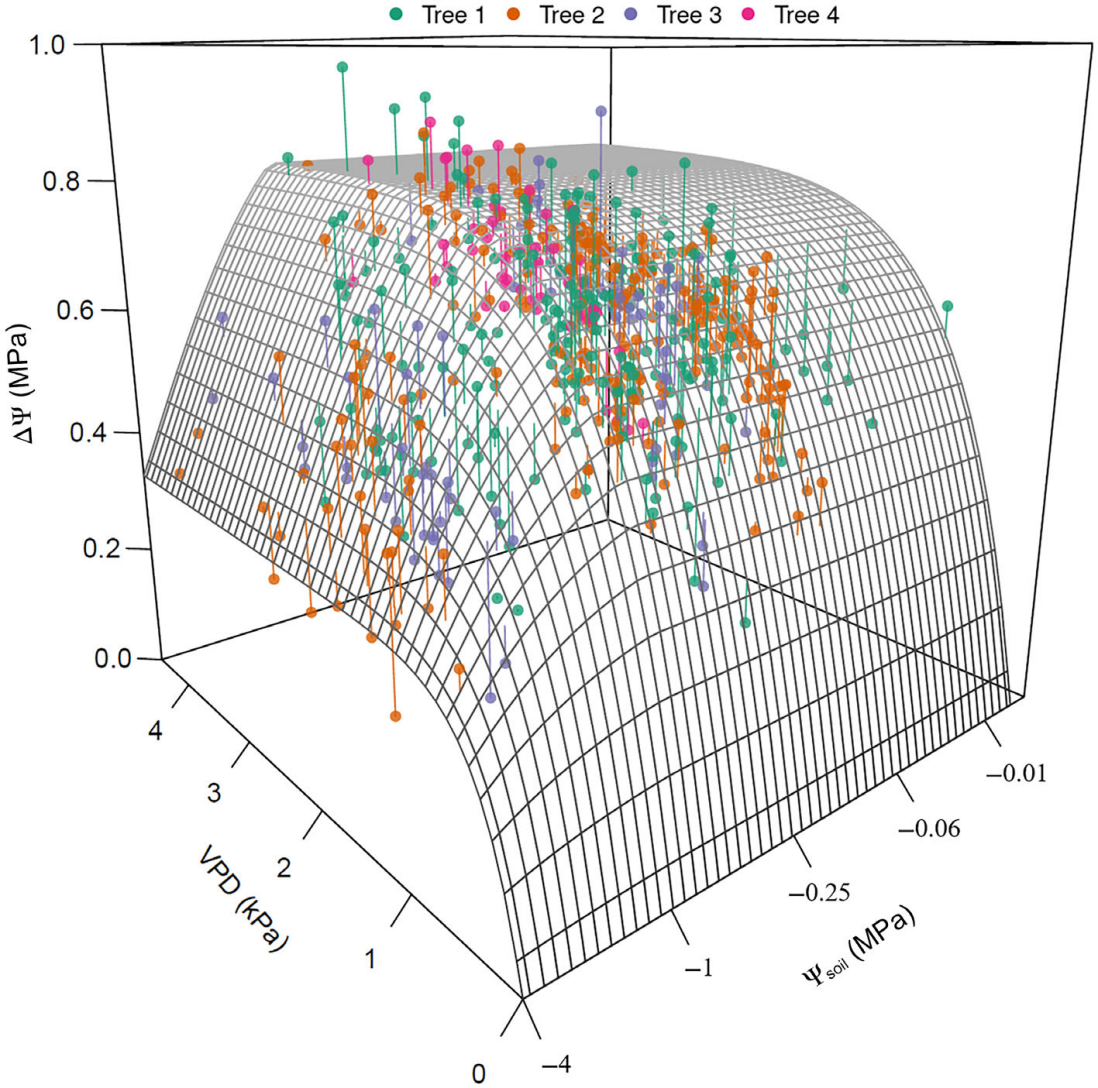
Three‐dimensional modelled surface representing the response of maximum daytime soil‐to‐stem water potential difference (ΔΨ) to vapour pressure deficit (VPD) and soil water potential (Ψ_soil_) in four trees of *Callitris rhomboidea* monitored across multiple highly variable growing seasons over a 4‐yr period between 2021 and 2024. The coloured lines depict the partial residuals with the corresponding coloured points showing the adjusted observations after accounting for plant‐ and year‐level effects. The axis for Ψ_soil_ is transformed to a log_4_ scale to reveal the spread of the observed data.

Under wet soil conditions (Ψ_soil_ close to 0 MPa), the modelled relationship, based on Eqns (Eqn 1), (Eqn 2), (Eqn 3), between daily maximum ΔΨ and VPD was characterized by an initial increase (at rate λ_0_) before approaching an asymptote (θ_0_), with ΔΨ attaining 95% of its asymptotic value when the VPD reached 1.94 kPa (1.53 and 2.67; 95% credible interval (CI)) (Figs 4a, 5). This trend was also observed in diurnal ΔΨ (Fig. 2a) and mirrors the commonly observed relationship between sap flux and VPD (Skelton *et al*., 2013; Grossiord *et al*., 2018, 2019), consistent with a hydraulic model whereby stomatal control exerts a limit on transpiration as leaf water potential decreases (Saliendra *et al*., 1995; Oren *et al*., 1999). This asymptotic relationship suggests that stomata were progressively closing as VPD increased, preventing further declines in Ψ_stem_, thus constraining ΔΨ outside the range of xylem embolism (Brodribb *et al*., 2020), but at the expense of reduced carbon gain (Saliendra *et al*., 1995; Bourbia *et al*., 2023).

### Fixed relationship between ΔΨ and VPD in the initial stages of soil drying

Examining the VPD relationship with ΔΨ over multiple and highly variable growing seasons enabled the effect of declining Ψ_soil_ on the VPD response to be examined. As the soil progressively dried (Ψ_soil_ became more negative), the relationship between ΔΨ and VPD initially remained constant (Fig. 5), as characterized by the constancy of θ and λ with declining Ψ_soil_ (Fig. 4b,c). This suggests that stomatal regulation of ΔΨ in response to VPD was insensitive to the initial decline in Ψ_soil_ and that the regulatory function that produced the asymptotic response between ΔΨ and VPD initially remained unchanged despite a decline in Ψ_soil_. Such apparent nonsensitivity of stomatal response to declining Ψ_soil_ might be attributed to a gradual shift in the osmotic potential of guard cells or mesophyll cells to more negative values during the initial stages of soil drying, a behaviour that has been observed in many species drying slowly in the field (Turner *et al*., 1978; McCree *et al*., 1984; Scholz *et al*., 2012; Cardoso *et al*., 2020) and described as isohydrodynamic behaviour (Franks *et al*., 2007). Maintaining a constant ΔΨ–VPD relationship may serve to maintain soil water extraction and extend carbon acquisition as the soil dries, but at the expense of declining Ψ_stem_ and risk of hydraulic damage (Franks *et al*., 2007). However, the maintenance of constant ΔΨ at high VPD by stomatal closure paradoxically restricts water uptake and carbon gain, which can place the plant at a disadvantage in competitive environments where water is limited.

### Changing relationship between ΔΨ and VPD below a Ψ_soil_ threshold

**Fig. 6:**
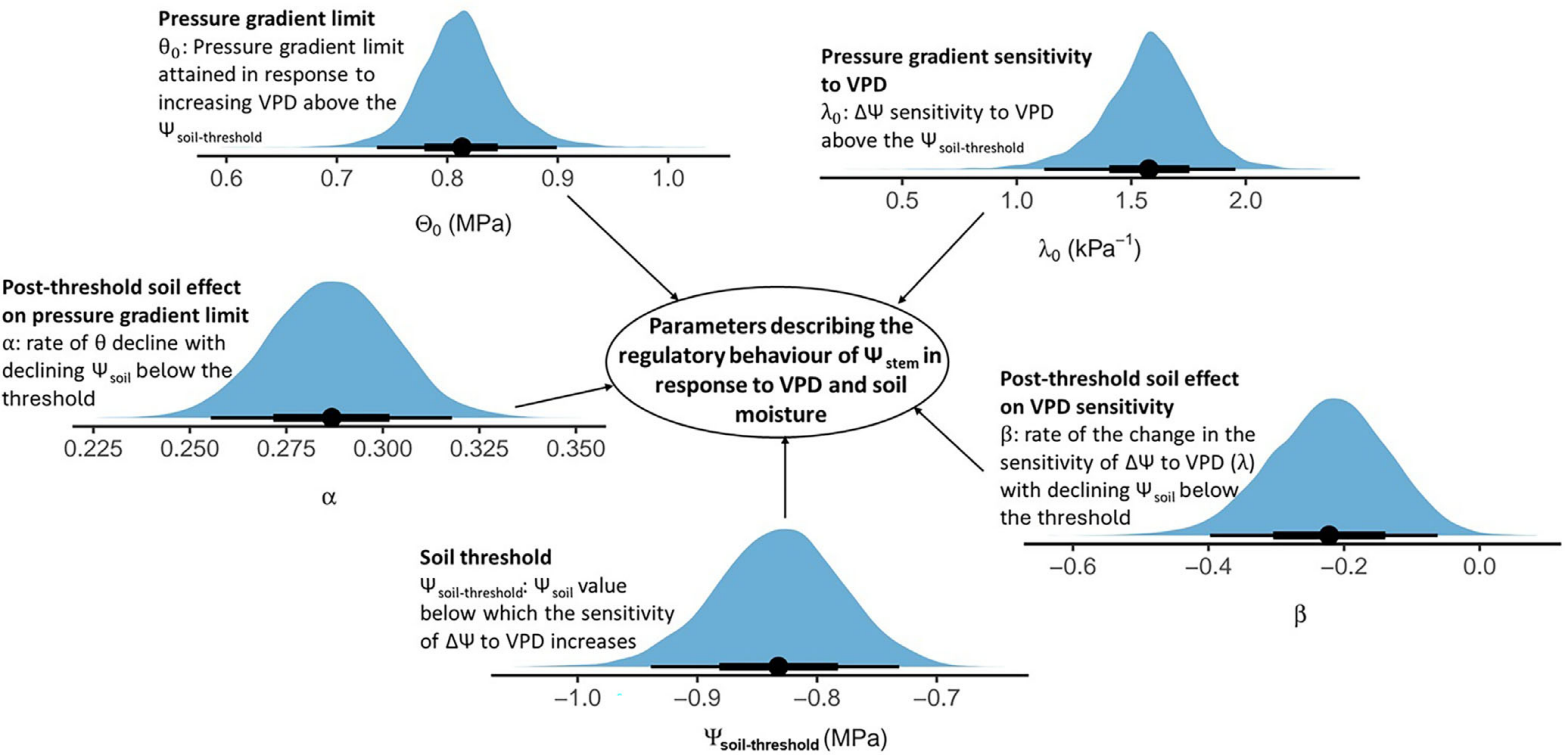
Posterior estimates of the five key parameters of the proposed hierarchical model for maximum daytime soil‐to‐stem water potential difference (ΔΨ) response to vapour pressure deficit (VPD) and soil water potential (Ψ_soil_) in *Callitris rhomboidea* monitored across multiple highly variable growing seasons between 2021 and 2024. The points are the posterior means, and the thick and thin lines are the 50% and 95% credible intervals, respectively. The blue shaded areas are the posterior densities.

Despite the strong conservation in the relationship between maximum daily ΔΨ and VPD under most levels of soil hydration, a clear threshold was observed as Ψ_soil_ decreased below −0.83 MPa (−0.939 and −0.731; 95% CI), whereupon all trees became highly sensitive to both Ψ_soil_ and VPD (Fig. 5). Beyond this threshold, a strong reduction in the ΔΨ asymptote (θ) (Fig. 5) with rate *α* = 0.29 (0.26 and 0.32; 95% CI) was observed. During this phase of strong limitation of ΔΨ, θ collapsed by > 50% as Ψ_soil_ reached −4 MPa (Figs 4b, 5). The sensitivity of ΔΨ to VPD also increased, with ΔΨ attaining the asymptote at progressively lower VPD as exhibited by the progressive increase in λ, with rate β = −0.22 (−0.4 and −0.06; 95% CI) (Fig. 6), as Ψ_soil_ declined below the threshold (Figs 4c, 5). These relationships indicate a pronounced stomatal regulation of Ψ_stem_ in response to both Ψ_soil_ and VPD and suggest a limit to further guard cell osmotic adjustment beyond this soil hydration threshold. Although the mechanism triggering such strong stomatal control below this Ψ_soil_ threshold remains unclear, we suggest that it may be caused by the triggering of abscisic acid production to strongly reduce guard cell turgor (Brodribb & McAdam, 2013; McAdam & Brodribb, 2014). The estimated Ψ_soil_ threshold appears to coincide with the value of Ψ_soil_ known to cause a steep decline in soil–plant hydraulic conductance (*K*_s‐p_) in this species (Bourbia *et al*., 2021), while also approximating the point where soil hydraulics become highly limiting for water extraction by plants (*c*. −1 MPa; Carminati & Javaux, 2020).

### Predictability of Ψ_stem_ regulation under seasonal changes in VPD and Ψ_soil_

Recent reports of Ψ_stem_ monitoring suggest that the temporal dynamics of ΔΨ in response to changes in Ψ_soil_ alone are highly unpredictable even within individuals of the same species between different seasons, casting doubt on the ability of Ψ_stem_ to characterize the behaviour of species (Guo *et al*., 2020, 2024; Kannenberg *et al*., 2022). Given that Ψ_stem_ is influenced by both VPD and Ψ_soil_, we assessed whether the regulatory behaviour of Ψ_stem_ can be accurately predicted across highly variable growing seasons when considering the combination of both physical factors. Our hierarchical model included data from different plants and years but allowed sufficient flexibility to accommodate plant and year‐specific variation (see the Materials and Methods section). Using this model, we found that 74% (71.6% and 75.5%; 95% CI) of the variance in maximum daily ΔΨ monitored in different individuals across highly variable growing seasons (Fig. 1) could be explained using only Ψ_soil_ and VPD as the dependent variables. This highlights the importance both these variables hold in modulating the water potential of the whole plant. We suggest that the lack of Ψ_stem_ predictability in previous studies using the existing metrics, such as isohydricity and hydroscape, might arise from a failure to consider VPD and its interaction with Ψ_soil_. The often‐reported inconsistency in Ψ_stem_ regulation as a function of Ψ_soil_ across different seasons of the same year is likely attributed to seasonal variation in VPD rather than alteration in stomatal behaviour (Guo *et al*., 2020, 2024; Kannenberg *et al*., 2022).

The generality of the model across different trees and seasons is supported by the lack of pattern with respect to plant when the residuals are plotted against both VPD and Ψ_soil_ (the mean and mean absolute error of the residuals were almost identical across plants; Fig. S4). The model's LOO *R* ^2^ is 0.72 (0.681 and 0.754; 95% CI), which is only slightly lower than the ordinary *R* ^2^ (0.74) (0.72 and 0.76; 95% CI), strongly suggesting that our statistical model is not overfit and has excellent ability to generalize to new data. In addition to the large percentage of variance explained, the validity of the model was demonstrated by the close correspondence between the observed and predicted data and the excellent calibration of the CIs (e.g. 96% of the data lie within the 95% CI; Fig. S5), as well as the model convergence diagnostic $\hat{R}$ (all values were < 1.003 where values must be below 1.01 for convergence).

**Fig. 7:**
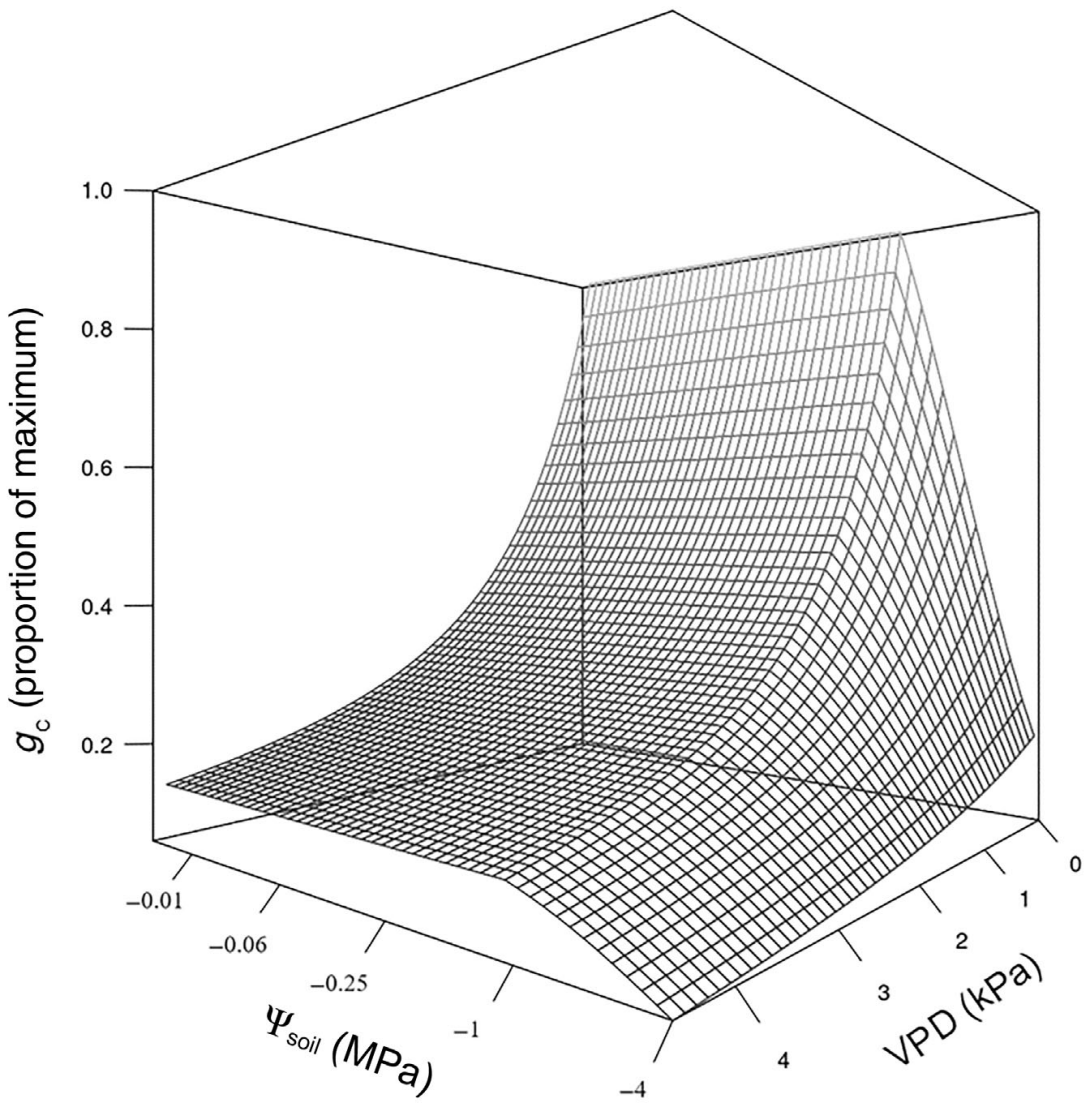
Surface response of whole plant diffusive conductance (*g*_c_) as a function of soil water potential (Ψ_soil_) and vapour pressure deficit (VPD) predicted from the modelled response of maximum daily soil‐to‐stem water potential difference (ΔΨ) to Ψ_soil_ and VPD in *Callitris rhomboidea* using a combination of Darcy's law and Fick's law of diffusion (Eqn 4). The axis for Ψ_soil_ is transformed to a log_4_ scale.

The five key parameters used to describe the association between ΔΨ and environmental variation provide a holistic characterization of a species water potential regulation in response to the joint effects of VPD and Ψ_soil_. These parameters should be readily and statistically comparable between species and genotypes. Furthermore, this water potential regulation can be readily translated to a stomatal phenotype (Fig. 7) given that stomatal behaviour is the primary driver of Ψ_stem_ regulation.

### Factors that might influence the predictability of Ψ_stem_ regulation in response to VPD and Ψ_soil_

It is worth pointing out that, in addition to VPD and Ψ_soil_, ΔΨ may also be influenced by other factors, such as changes in soil water viscosity due to fluctuation in soil temperature, especially at the seasonal time scale (Lintunen *et al*., 2020). In this study, we measured soil temperature in 2024 at the depth of 0.3 m and it was found to vary by 3°C throughout the whole growing season (between December 2023 and April 2024); hence, it may have a small impact. Additionally, changes in root : shoot ratio or xylem hydraulic conductance due to plant developmental and phenological changes or acclimation to new climates, which might occur over long term, such as throughout the growing season or across different years, have the potential to alter the whole plant hydraulic conductance. This can influence stomatal sensitivity to VPD and soil moisture and, consequently, the regulatory behaviour of Ψ_stem_ (Marchin *et al*., 2016; Anderegg, 2018; Sharma *et al*., 2024). However, the lack of significant difference in θ_0_ and λ_0_ above the Ψ_soil_ threshold (under relatively hydrated conditions) within individuals across different years (Fig. S6) suggests that root : shoot ratio and xylem conductivity are somewhat conserved in this species, but the possibility of changes occurring throughout a growing season or over multiple years remains. Nevertheless, the inclusion of these additional factors might help to reduce the remaining small portion of unexplained variance in our model; hence, it is important to develop methods to account for their effects in future studies. Other factors, such as changes in air CO_2_ concentration, light interception and extreme temperature, can also influence stomatal behaviour and the resulting relationship between Ψ_stem_, VPD and soil moisture. Therefore, accounting for these drivers in the model proposed in this study is also important for improving the predictability of Ψ_stem_. In this study, the VPD data used for studying the regulation of Ψ_stem_ were collected from a single sensor attached to one of the measured trees, positioned in the middle of the tree canopy. However, VPD within the canopy may vary slightly between trees, especially if they differ in canopy density. Using only one sensor could therefore introduce some variability in ΔΨ–VPD relationship between trees. Placing a sensor on each tree where Ψ_stem_ is being measured will minimize this potential variability and improve the accuracy of the model.

It is worth also noting that the model was tested on trees at the same site growing in the same soil type (well‐drained clay loam). However, different soil types are known to have varying hydraulic conductances, which may respond differently to soil drying, potentially influencing stomatal behaviour and water potential regulation to varying extents (Koehler *et al*., 2024; Wankmüller *et al*., 2024). Therefore, whether the within‐species predictability of water potential regulation remains consistent across different soil types is unclear and requires further investigation. The same uncertainty applies to sites with differing climates (e.g. dry vs wet sites, elevation), where the same species may exhibit different adaptations and water use strategies (Feng *et al*., 2019). In this study, however, the observed consistency in ΔΨ behaviour across the markedly different seasons between different individuals suggests that the model parameters may also be conserved across different environmental gradients in the studied species.

### Predicting whole plant diffuse conductance responses to VPD and Ψ_soil_ from water potential regulation

Several models have been proposed to predict *g*_c_ response to changing environmental conditions (Ball *et al*., 1987; Leuning, 1990; Medlyn *et al*., 2011; Buckley & Mott, 2013; Sperry *et al*., 2016; Wang *et al*., 2020; Cochard *et al*., 2021). However, the formulation of the Ψ_soil_ and VPD effects in these models is generally empirical (Ball *et al*., 1987; Leuning, 1990) or theoretical (Franks & Farquhar, 1999; Sperry *et al*., 2016; Wang *et al*., 2020). Given that stomata respond to VPD and Ψ_soil_ through changes in leaf water potential, one promising way to improve current models is to directly link stomatal behaviour to water potential (Buckley *et al*., 2012). This approach should provide a more realistic basis for predicting stomatal responses as long as the parameterization describing species‐specific relationships between Ψ_stem_ and *g*_c_ can be defined. Here, we inferred whole plant stomatal responses to the combined effect of VPD and Ψ_soil_ using the parameters derived from our statistical model, which describes the regulation of ΔΨ to VPD and Ψ_soil_ (Fig. 7). Under relatively hydrated conditions (Ψ_soil_ close to 0 MPa), whole plant stomatal diffusive conductance (*g*_c_) was predicted to decline exponentially and by > 80% as VPD increases to 4 kPa, a behaviour reported in several species under wet conditions (Oren *et al*., 1999; Novick *et al*., 2016; Grossiord *et al*., 2018). As Ψ_soil_ declined due to soil drying, *g*_c_ maintained the same response relationship with VPD and showed no sensitivity to this decline until Ψ_soil_ reached −0.83 MPa. Below this Ψ_soil_ threshold, stomata became highly sensitive to VPD and closed to a greater extent thereafter (Fig. 7). This suggests that, in this species, VPD can be the main driver of carbon gain and water loss across the range of soil moisture conditions experienced during most of the growing season during average years, such as 2023, where Ψ_soil_ never declined significantly below the threshold (Fig. 1) (Ψ_soil_ between 0 and −1 MPa). We point out that this prediction assumed constant *K*_s‐p_ during drying; however, applying the *K*_s‐p_ response to Ψ_soil_ observed in potted *C. rhomboidea* plants (Bourbia *et al*., 2021) would only increase the slope of the stomatal closure response below the Ψ_soil_ threshold.

Existing optimization models posit that stomata close to minimize the cost of reduction in within or outside xylem hydraulic conductance caused by low water potential (Sperry & Love, 2015; Sperry *et al*., 2016; Wolf *et al*., 2016; Anderegg *et al*., 2018; Joshi *et al*., 2022). On the other hand, soil hydraulic models suggest that stomatal closure is optimal when it corresponds with steep declines in the rhizosphere hydraulic conductance (Carminati & Javaux, 2020). Both models, however, predict that stomata should remain open regardless of the change in Ψ_soil_ or VPD if the risk of reduction in soil–plant hydraulic conductance is negligible. However, the theory behind these models does not perfectly align with our data, showing a strong decline in *g*_c_ at high VPD even in well‐hydrated soils (Ψ_soil_ close to 0 MPa) where no decline in *K*_s‐p_ is expected in this species (Bourbia *et al*., 2021). This is further supported by evidence from a recent study reporting stomata to close at high VPD under ample soil moisture without any decline in *K*_s‐p_ for the species studied here (Bourbia & Brodribb, 2024). On the other hand, the Ψ_soil_ trigger point for aggressive stomatal closure is rather close to the point of declining *K*_root_ for the studied species *C. rhomboidea* (Bourbia *et al*., 2021) and the prediction for optimal soil water extraction (Carminati & Javaux, 2020). Therefore, the behaviour of *g*_c_ in *C. rhomboidea*, specifically under drying soil, does appear consistent with optimal regulation of transpiration to protect the xylem and to optimally extract water from the soil.

### Conclusion

Long‐term monitoring of Ψ_stem_ in four trees revealed a highly predictable regulatory behaviour of xylem water potential and inferred stomatal conductance during highly variable growing seasons over 4 yr. Future work will confirm the application of this approach to characterize diverse species with varying behaviours across different environmental gradients, such as varying soil types and climates. Nevertheless, this characterization of a species' water use physiology opens the way for wider comparisons between genotypes and species, using a common monitoring and statistical protocol. The resultant potential for phenotypic screening in crop species is highly significant, while the ecological application for understanding and characterizing diversity among species in water use ‘strategy’ will enable a deeper understanding of how plants compete and survive during seasonal variation in water availability.

## Competing interests

None declared.

## Author contributions

IB and TJB planned and designed the research. IB and TJB performed experiments. IB, LAY and TJB analysed data. IB, LAY and TJB wrote the manuscript.

## Disclaimer

The New Phytologist Foundation remains neutral with regard to jurisdictional claims in maps and in any institutional affiliations.

## Supporting information


**Table S1** Time‐series of stem water potential (Ψ_stem_) analysed in this study.

**Fig. S1** Relationship between branchlet width and Ψ_stem_.
**Fig. S2** Diurnal changes in VPD, Ψ_stem_ and Ψ_soil_.
**Fig. S3** Response of diurnal changes in ΔΨ to diurnal VPD.
**Fig. S4** Distribution of the modelled ΔΨ residuals against VPD and Ψ_soil_.
**Fig. S5** Comparison between the observed and predicted ΔΨ.
**Fig. S6** Posterior estimates by plant and year for the asymptote θ_0_ and rate λ_0_.
Please note: Wiley is not responsible for the content or functionality of any Supporting Information supplied by the authors. Any queries (other than missing material) should be directed to the *New Phytologist* Central Office.

## Data Availability

The raw data that support the findings of this study are available in Dataset S1.
