# Supplementary material for: Using long‐term field data to quantify water potential regulation in response to VPD and soil moisture in a conifer tree
Source: New Phytol. 2025 Mar 13;246(3):911–23. doi: 10.1111/nph.70056 (PMC11982795; doi:10.1111/nph.70056)
Supplement: Supplementary file 2 — Fig. S1 Relationship between branchlet width and Ψstem. Fig. S2 Diurnal changes in VPD, Ψstem and Ψsoil. Fig. S3 Response of diurnal changes in ΔΨ to diurnal VPD. Fig. S4 Distribution of the modelled ΔΨ residuals against VPD and Ψsoil. Fig. S5 Comparison between the observed and predicted ΔΨ. Fig. S6 Posterior estimates by plant and year for the asymptote θ0 and rate λ0. Please note: Wiley is not responsible for the content or functionality of any Supporting Information supplied by the authors. Any queries (other than missing material) should be directed to the New Phytologist Central Office. [file NPH-246-911-s002.docx]

**New Phytologist Supporting Information**

Article title: Using long term field data to quantify water potential regulation in response to VPD and soil moisture in a conifer tree

Authors: Ibrahim Bourbia, Luke A. Yates, Timothy J Brodribb

Article acceptance date: 6 February 2025


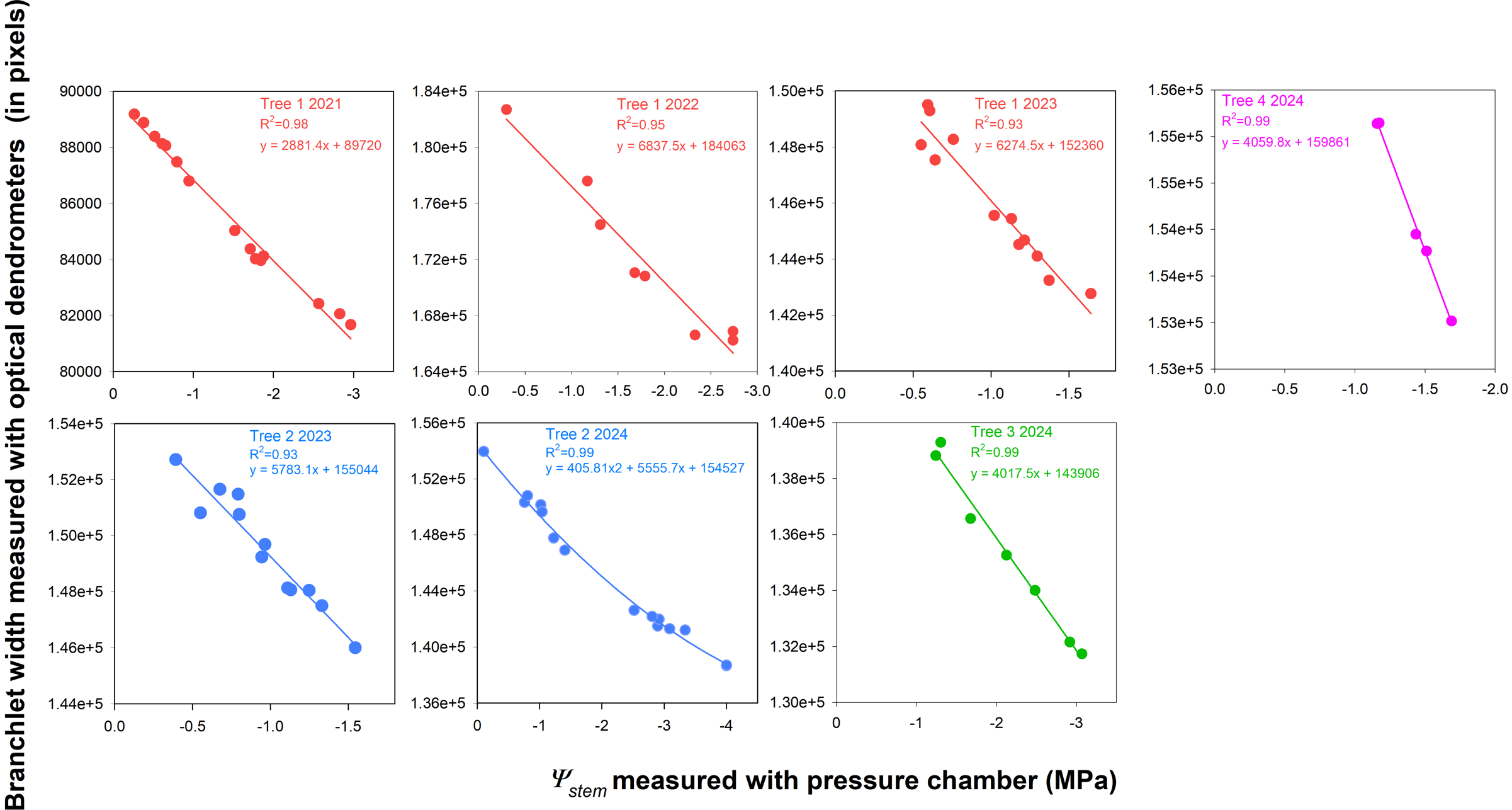


**Fig. S1:** Relationship between branchlet width monitored in the field with optical dendrometry and corresponding stem water potential (*Ψ_stem_*) measured on neighbouring covered non-transpiring branchlets with a pressure chamber in four trees of *C. rhomboidea* over multiple growing seasons between 2021 and 2024. *Ψ_stem_* exhibited a strong relationship with branchlet width in all trees across each growing season *R*^2^ (> 0.93). The relationship between *Ψ_stem_* and branchlet width was linear in all trees, expect in tree 2 (2024) which was quadratic.

**
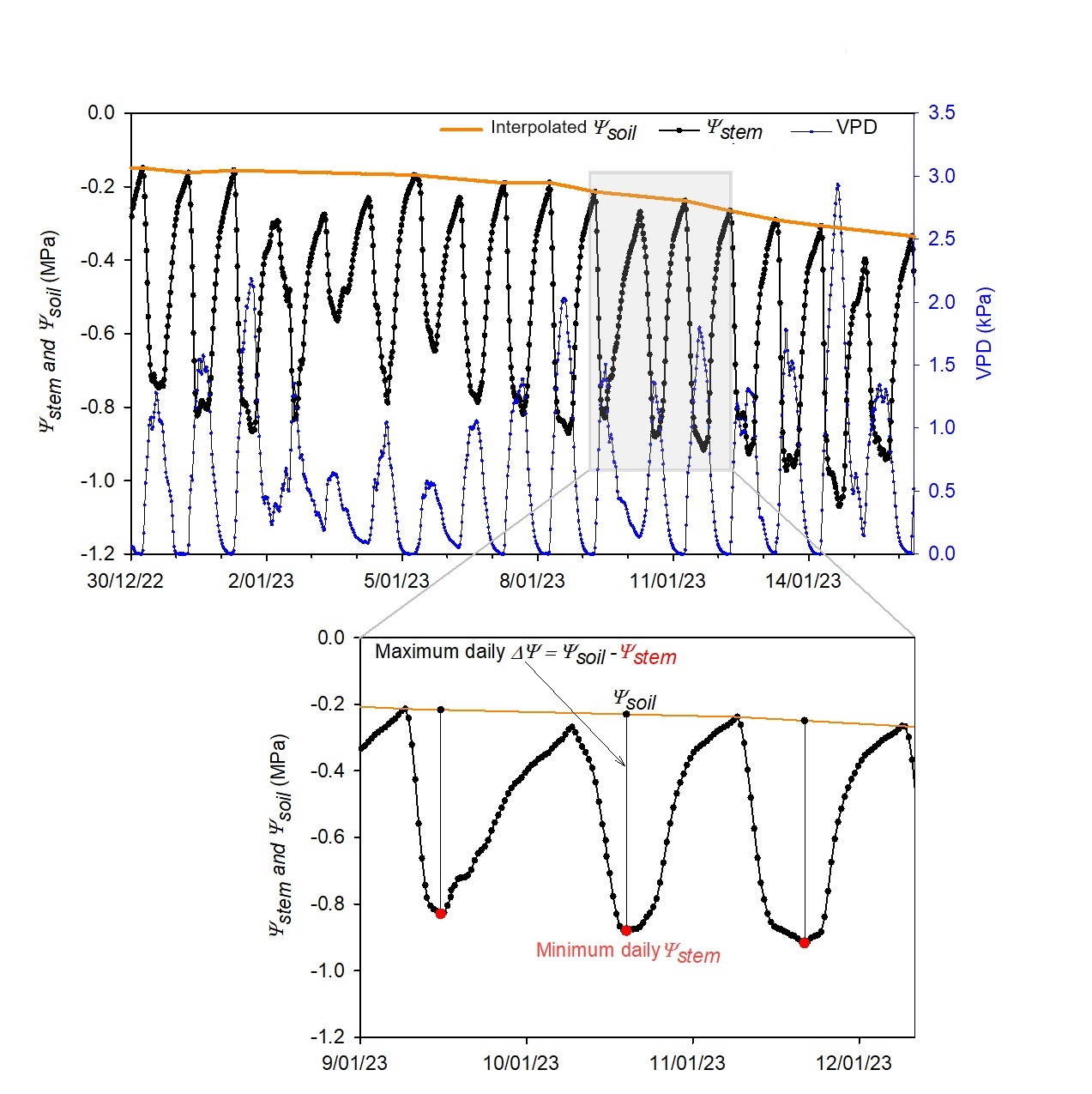
**

**Fig. S2:** Zoomed-in view of diurnal changes in vapour pressure deficit (VPD), stem water potential (*Ψ_stem_*) and interpolated soil water potential (*Ψ_soil_*) over a few days in one tree of *C. rhomboidea*. In each individual tree, maximum daily soil-to-stem water potential difference (*ΔΨ*) was calculated as the difference between minimum mean daily *Ψ_stem_* (most negative value) averaged over a 2-hour period (using 4-8 datapoints) and its corresponding daytime *Ψ_soil_*. The corresponding daytime *Ψ_soil_* throughout the study period for each individual tree was determined using linear interpolation between the optically derived predawn *Ψ_stem_* values (maximum value of *Ψ_stem_* before sunrise averaged over 2 hours). Predawn *Ψ_stem_* can be strongly influenced by night-time VPD, and this might potentially influence the magnitude *ΔΨ* and its relationship with VPD and *Ψ_soil_.* For this reason, the predawn *Ψ_stem_* values used for interpolating *Ψ_soil_* throughout the study period in each individual tree were selected on the nights where humidity remained above 95% (VPD <0.05 kPa) throughout the whole night (between midnight and before sunrise). Under such conditions, night-time transpiration was found to be negligible in this species, hence predawn *Ψ_stem_* was assumed to have been given sufficient time to establish equilibrium with *Ψ_soil_* in the rooting zone.

**
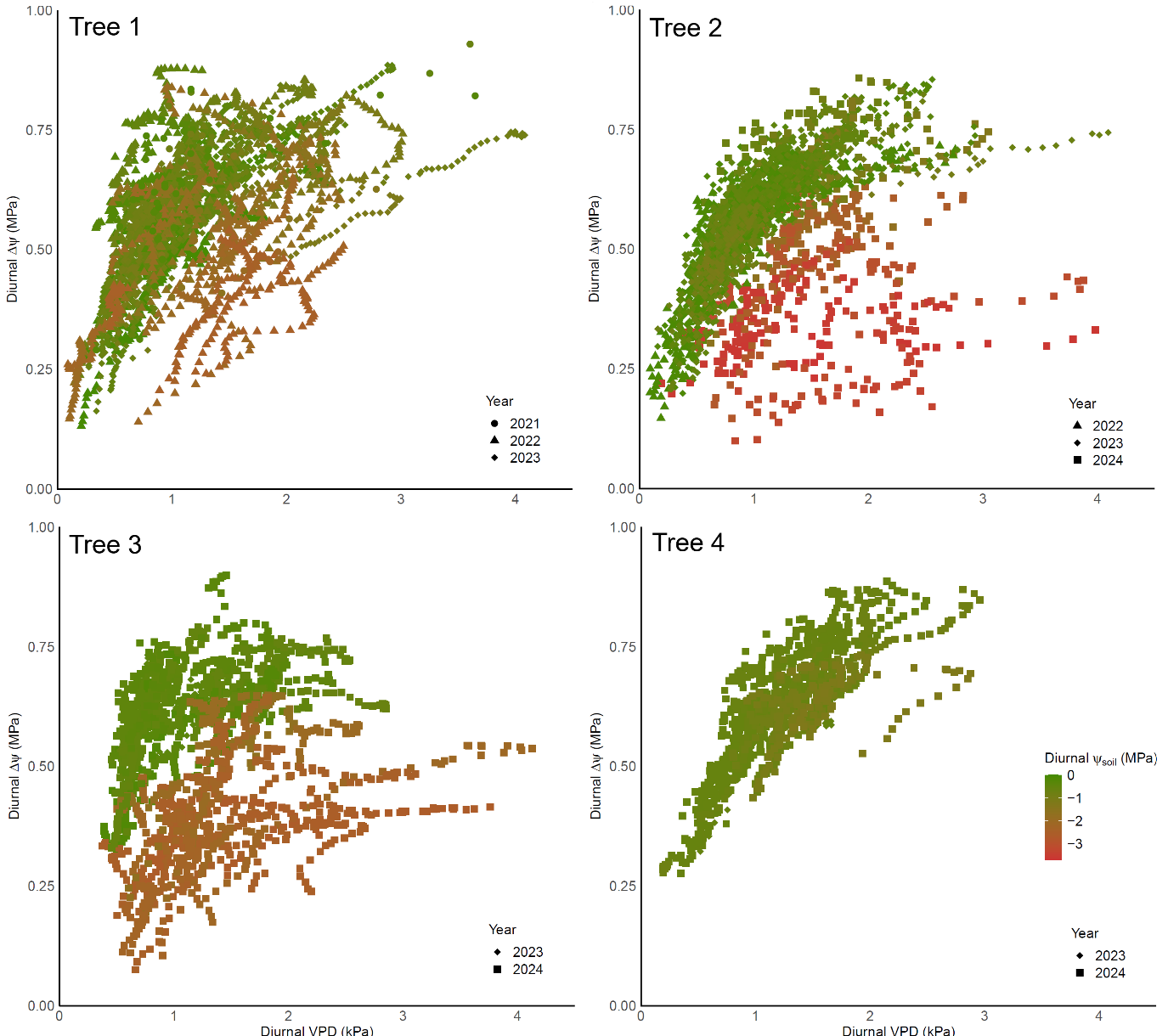
**

**Fig. S3:** Response of diurnal changes in maximum daytime soil-to-stem water potential difference (*ΔΨ*) to corresponding changes in diurnal vapour pressure deficit (VPD) and soil water potential (*Ψ_soil_*) between 11:00h and 16:00 h in four trees of *C. rhomboidea* monitored over multiple highly variable growing seasons over a four-year period between 2021 and 2024.


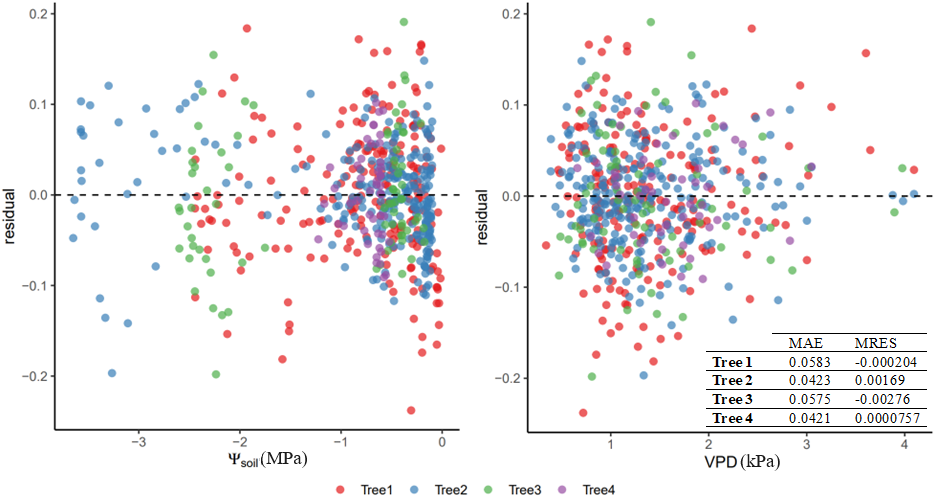


**Fig. S4:** Distribution of the modelled maximum daytime soil-to-stem water potential difference (*ΔΨ*) residuals against both vapour pressure deficit (VPD) and soil water potential (*Ψ_soil_*) in four trees of *C. rhomboidea* monitored across multiple growing seasons and diverse seasonal conditions of VPD and *Ψ_soil_* between 2021 and 2024.  The residuals show no apparent pattern. The mean (MRES) and mean absolute error (MAE) values of the residuals were almost identical across trees (inset table).


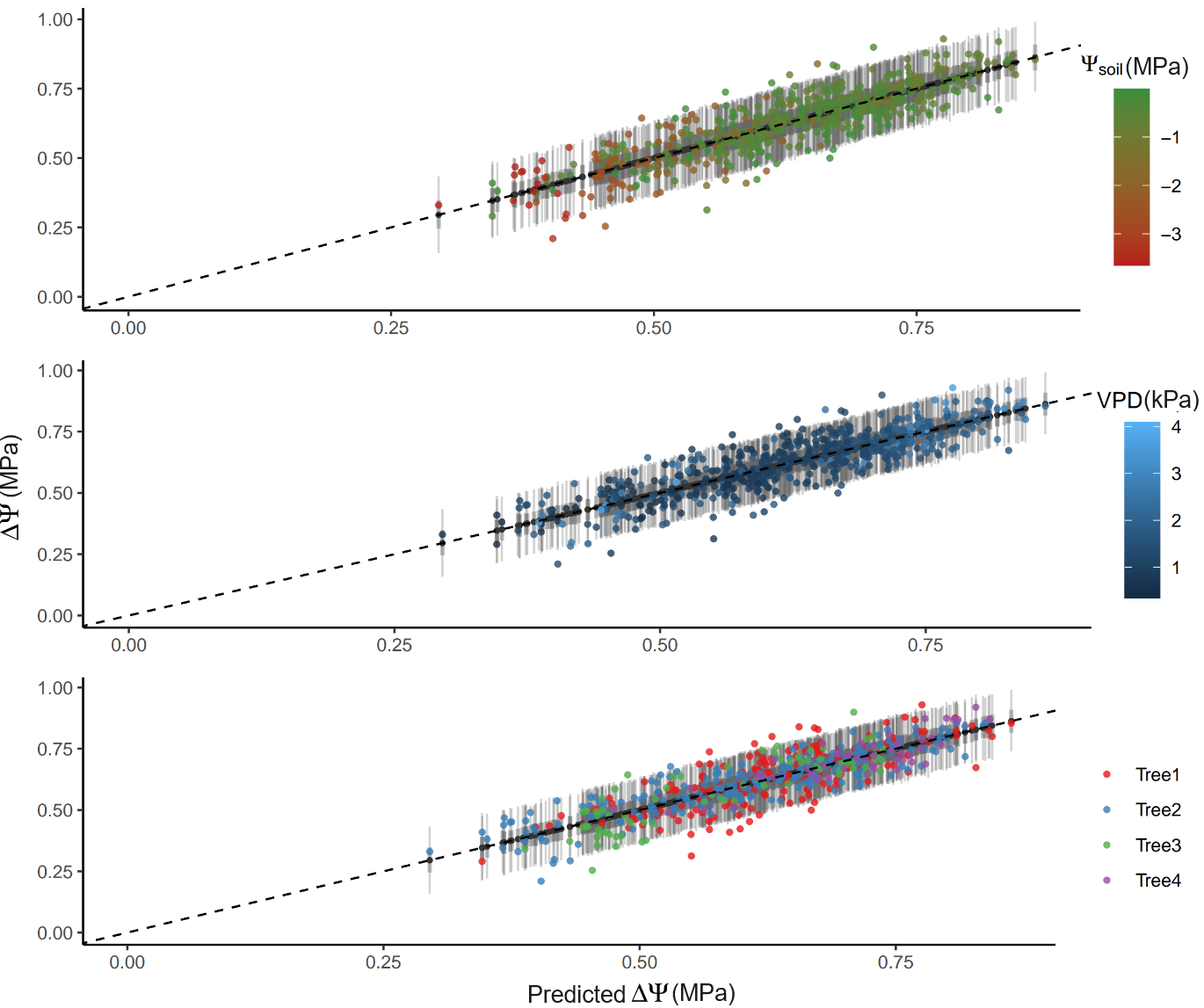


**Fig. S5**: Comparison between the observed and predicted maximum daytime soil-to-stem water potential difference (*ΔΨ*) values in four trees of *C. rhomboidea* monitored across highly variable growing seasons between 2021 and 2024. The coloured points are the observed values versus the predicted means. The black points are the predicted means, and the thick and thin grey vertical lines are the 50% and 95% posterior predictive intervals, respectively. The observations are ordered by increasing predicted mean coloured by soil water potential (*Ψ_soil_*), vapour pressure deficit (VPD) and tree. The dashed line represents the 1:1 line. 96% of the observed *ΔΨ* data lie within the 95% predictive interval.

**
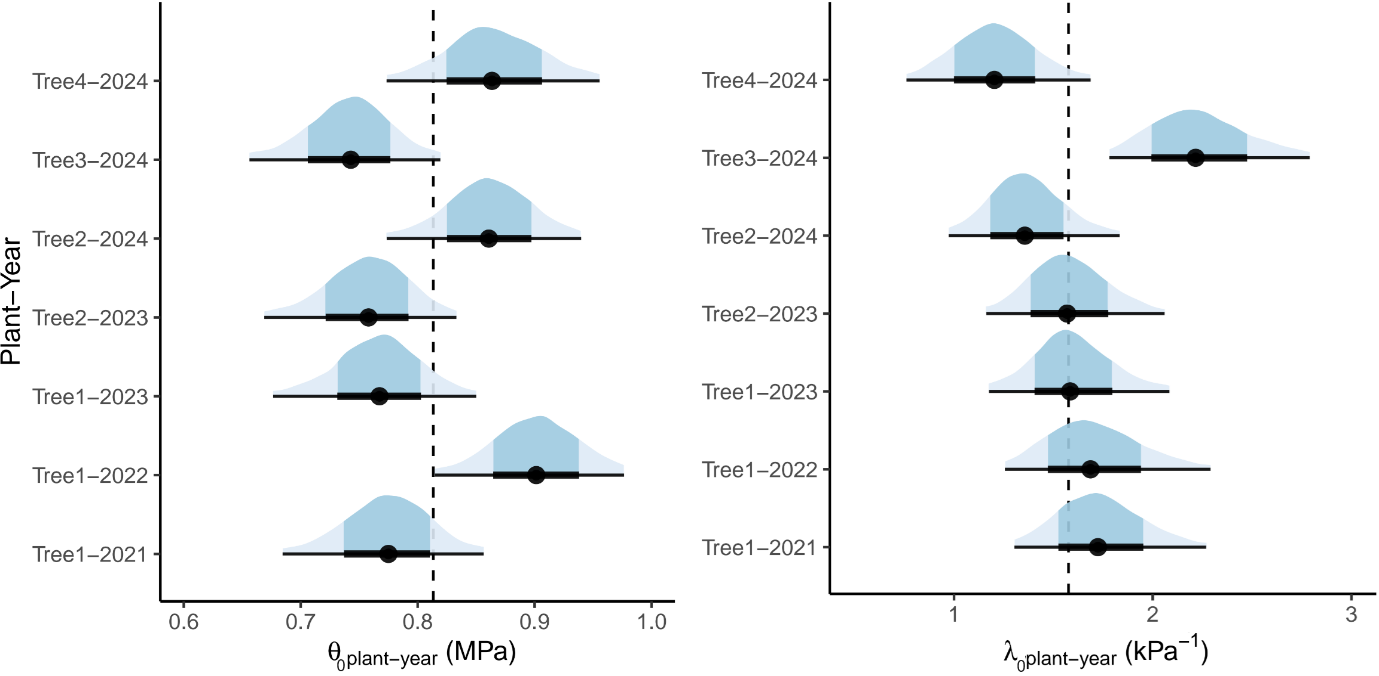
**

**Fig. S6:** Posterior estimates by plant and year for the asymptote θ_0_ and rate λ_0_ under relatively hydrated soil (before the *Ψ_soil_threshold_*) in four trees of *C. rhomboidea* monitored over multiple growing seasons. The points are the posterior means, and the thick and thin lines are the 50% and 95% credible intervals, respectively. The dashed line is the mean estimate of the parameters.
